# “I do all I can but I still fail them”: Health system barriers to providing Option B+ to pregnant and lactating women in Malawi

**DOI:** 10.1371/journal.pone.0222138

**Published:** 2019-09-12

**Authors:** Nozgechi Phiri, Kali Tal, Claire Somerville, Malango T. Msukwa, Olivia Keiser

**Affiliations:** 1 Institute of Global Health, University of Geneva, Geneva, Switzerland; 2 Baobab Health Trust, Lilongwe, Malawi; 3 Institute of Primary Health Care (BIHAM), University of Bern, Bern, Switzerland; 4 Gender Centre, Graduate Institute, Geneva, Switzerland; Johns Hopkins Bloomberg School of Public Health, UNITED STATES

## Abstract

Malawi’s Option B+ program is based on a ‘test and treat’ strategy that places all HIV-positive pregnant and lactating women on lifelong antiretroviral therapy. The steep increase in patient load placed severe pressure on a health system that has struggled for decades with inadequate supply of health care workers (HCWs) and poor infrastructure. We set out to explore health system barriers to Option B+ by asking HCWs in Malawi about their experiences treating pregnant and lactating women. We observed and conducted semi-structured interviews (SSIs) with 34 HCWs including nine expert clients (ECs) at 14 health facilities across Malawi, then coded and analyzed the data. We found that HCWs implementing Option B+ are so overburdened in Malawi that it reduces their ability to provide quality care to patients, who receive less counseling than they should, wait longer than is reasonable, and have very little privacy. Interventions that increase the number of HCWs and upgrade infrastructure to protect the privacy of HIV-infected pregnant and lactating women and their husbands could increase retention, but facilities will need to be improved to support men who accompany their partners on clinic visits.

## Introduction

Malawi’s Option B+ program is a ‘test and treat’ strategy that targets pregnant and lactating women. Starting in 2011, women who present for antenatal care (ANC) are tested and, if found HIV-positive, begin lifelong antiretroviral therapy (ART) without the need for CD4 count or clinical staging. Malawi adopted Option B+ to simplify ART initiation for HIV infected pregnant and lactating women considering the limited CD4 count capabilities in the health facilities. The aim of Option B+ was to reduce new HIV infections in children and promote good maternal health. [1,2] Option B+ was integrated into existing maternal, newborn and child health services [3,4], including antenatal and postpartum care, family planning, treatment of sexually transmitted infections, and cervical cancer screening. Within a year, Option B+ had more than doubled the number of HIV-positive pregnant and lactating women on ART [4–6], from 22% to 47%. [6] Malawi Population- based HIV Impact Assessment (MPHIA) estimated an HIV prevalence of 8.7% for pregnant women aged 15–49 years between 2015 and 2016. [7] While ‘test and treat’ is now the policy that the WHO recommends [8], and its effectiveness has been proven, the decision of nations to target women and children in ANC may have unexpected effects, including endangering women [9,10] and sidelining men. [11] Our earlier research showed that Malawian women stay on ART because they believe it protects their own and children’s health, [12] but in an overburdened health care system, attendance presents challenges even to motivated women. [13–17]

Option B+ was adopted and implemented in a health system meant to serve a smaller population. [2] The steep increase in patient load placed severe pressure on a health system that has struggled for decades with inadequate supply of health care workers (HCWs) and poor infrastructure. [2,18] Health facilities in Malawi operate at a 45% vacancy rate, [19] so even the most committed staff members often work under impossible conditions and cannot meet their obligations to serve all their patients or even, sometimes, keep their temper. Inadequate supply of HCWs has been associated with loss-to-follow-up (LTFU) [20,21], as have other related factors like inadequate initial counseling [13,20,21], long waiting times and bad attitude from HCWs [5,14,22]. Poor infrastructure also increases the risk that HCWs will violate patients’ privacy and confidentiality, which can also limit attendance. [23] But few studies have asked HCWs how their working conditions affect patients’ quality of care, or how HCWs meet the challenges they face. We set out to explore health system barriers to Option B+ by asking HCWs in Malawi about their experiences treating women in their care.

## Methods

### Study setting and population

Our study was conducted to complement our earlier studies on Option B+ program in Malawi. [24,25] We selected and collected data from 14 districts from the list of our previous sites in the central and southern regions of Malawi, and included 11 district hospitals, one central hospital, and two health centres. We conducted interviews and stopped recruitment after we reached saturation.

We visited the health facilities and interviewed HCWs in the Option B+ program, focusing on nurses and expert clients (ECs) at ANC and ART clinics. We interviewed ECs who support nurses in providing health education, pre- and post-HIV/ AIDS counselling, perform initial clinical measurements (weight, height, blood pressure), archiving patient records, collect infant dried blood samples, or dispensed antiretroviral drugs (ARVs). ECs were present in eleven of the health facilities we visited. In two of the health facilities, ECs collected dried blood samples in line with the Malawian HIV testing services guidelines that allows volunteers to conduct HIV testing and counseling (HTC) after completing a certified HTC course. [26] We received permission from each hospital management team to collect data. We explained our study aims and process to the nurse in- charge of the ANC or ART department who allowed us to observe and interview HCWs in their department. We approached potential participants individually, explained to them our study information, procedure and obtained consent (see details under ethical considerations). We were especially interested in interviewing HCWs active in the Option B+ program at the time of our visit. The number of HCWs varied from site to site and so did their availability for interviews.

### Data collection

We collected data between November 2014 and December 2015, combining SSIs of HCWs with ethnographic observations at health facilities. Interviews were based on an interview guide. The first set of questions allowed the interviewer to build rapport. The interviewer asked each HCW to describe in detail their role in Option B+ and how well they felt they were able to execute their role. These first questions gave the interviewer the opportunity to ask specific questions about the challenges HCWs faced in providing Option B+, including asking participants to cite examples. The interviewer sometimes referred to observations, offering them as a bridge so a HCW could respond to and expand upon them.

Ethnographic observations focused on the facility, individuals, and interpersonal interactions. Our observations were based on a guide. We paid attention to the structure of the facility and the sections that provided Option B+, noting down information about space, number of staff and patients, and availability of equipment and drugs. We observed each step of patients’ clinic visits to identify differences between initial or subsequent ANC visits, waiting time, total time spent at the facility, and provider-client interactions. We sometimes informally asked HCWs questions when we needed more context about what we observed. We took light notes during the observations, which we expanded into detailed field notes soon after leaving the health facility. We did not collect confidential or identifying information during the observations. We scheduled one observation at each facility on the first day of the site visit, we continued the next day only when we failed to capture all the activities on the first day. We followed the procedures as they occurred and participants as they worked at the health facility but did not take part in any of the procedures.

When HCWs agreed to be interviewed, the interviewer let the HCWs choose the time. Most HCWs were interviewed in their offices, but sometimes the interviewer had to improvise, and a few interviews were conducted in the project car when no room was available to maintain privacy. To maintain confidentiality and a safe environment, there were no external observers present during the interviews. Interviews were conducted in Chichewa, the local language, or English, or in both languages. Interviews were recorded on a Sony IC recorder and transcribed verbatim in Chichewa, then later translated to English for coding.

### Data analysis

A team of five trained transcribers transcribed the interviews and our local researcher, checked the transcripts against the media files. We uploaded all transcribed English transcripts and observation notes into ATLAS.ti, qualitative software, and thematically analyzed the data. Thematic analysis has been described as “a method of identifying, analyzing and reporting emerging patterns within the data”. [27] We chose the well-established method of reflexive thematic analysis described by Braun et al. [28], because this method presumes meaning is a product of context, and that subjectivities (e.g., familiarity with culture and language) were resources that added value to our observations and analysis. In line with this method, we did not pre-specify themes or categories; they arose organically out of our analytic work. We took an inductive approach, where our local researcher, Nozgechi Phiri (NP), read and reread the transcripts and then developed initial codes based on our research questions. Another researcher, Kali Tal (KT), reviewed the codes and the transcripts. We added and refined the codes and emerging themes and subthemes as we further reviewed the transcripts. NP, KT, and Claire Somerville (CS) discussed the emerging themes before presenting and reporting. We resolved any disagreements by consensus.

### Ethical considerations

In Malawi, we obtained ethical approval for the study from the National Health Sciences Research Committee (NHSRC), approval number NHSRC 1284. In Switzerland, the Ethics Committee waived the requirement for ethical approval, req-2016-00090. We included only HCWs who gave written informed consent. Participants were informed that their participation was voluntary and therefore they can refuse or withdraw their participation at any point without penalty. We asked participants for verbal consent to record the interviews. Recorded interviews were de-identified. We went through the interviews and made sure the transcripts did not contain personal identifying information. Participants were given money to cover their lunch because our interviews were scheduled after clinic hours.

## Results

We interviewed 34 HCWs, most of whom were nurses (n = 25) Table 1. Most were women (n = 29), and qualified nurse- midwife technicians (n = 11) or registered nurses (n = 8). Of the nurses, five were managers: four registered nurses (RN) and one nurse- midwife technician (NMT). We also interviewed nine ECs. The median age of the HCWs was 36.5 years (interquartile range: 28–57).

**Table 1:** Health care workers' characteristics (N = 34).

	N (%)
Gender	
Female	29 (85.3)
Male	5 (14.7)
Age (years)	
20–29	5 (14.7)
30–39	15 (44.1)
40–49	10 (29.4)
>50	4 (11.8)
Median (IQR*)	36.5 (28–57)
Marital status	
Married	21 (61.8)
Divorced	3 (8.8)
Widowed	7 (20.6)
Single	3 (8.8)
Qualification	
Registered nurse/ Midwives	8 (23.5)
Nurse midwife technician (NMT)	11 (32.4)
Community nurse/Midwives	6 (17.7)
Expert client	9 (26.5)

*IQR: Interquartile range

### Observations

This section summarize our findings from observations conducted at each facility. Specific observations are integrated into the thematic categories, below. We saw crowds of women attending ANC, and crowds of men and women at ART clinics and in some of the clinics, they did not have enough benches to sit everyone such that some women sat on the floor. We watched HCWs working, saw how overburdened they were. In most sites, HCWs took care of large groups of women with limited personnel. In other sites with smaller groups of women HCWs were overburdened with multiple tasks like antenatal care, family planning etc. Having multiple roles was common across the sites. We watched as one nurse tended to a maximum of 72 women on her own at an ANC clinic. In some days, we observed fewer as 15 women but on average HCWs saw 60 women. On some days, clinics had no HCWs to perform HIV tests; in others, HIV testing or services in general started late. We saw that on their initial visits women spent more time at the clinic than those on their later visits. The need for more space or rooms in most of the ANC clinics was clearly apparent, since HCWs used one room for several services like ANC, Prevention of Mother-to-Child Transmission (PMTCT) or child services. At one clinic, we observed 14 people (including a couple) being offered services in one room by different HCWs, breaching the confidentiality of the clients. At some ART clinics, we saw nurses admit three clients (men and women) into one room because they had only one room for three nurses. We observed how few men attended ANC with their wives, spotting at most eight couples in a day, while on some days there was none. ARVs were in stock at all clinics.

### Semi structured interviews (SSI)

We analysed the data until four themes emerged. HCWs felt they had trouble carrying out the tasks required to support the Option B+ program for four reasons: 1) staff shortages; 2) inadequate infrastructure; 3) clinics were not organized to encourage male attendance, and 4) culture and gender interfered with acceptance and adherence to ART. Staff shortages affected a) the quality of counselling, b) HCW- client relationships, and c) lengthened clients’ visits to the health facility. Inadequate infrastructure put HCWs in the position of unwillingly disclosing clients’ HIV status. HCWs thought men were dissatisfied with how clinics were organized, and therefore gave preference to men so that they would remain and return with their partners. They also observed that culture and gender affected women’s acceptance of HIV care.

### Staff shortage

Most of the HCWs (27/35) we interviewed thought understaffing in the clinics was the main challenge they faced while serving women on or about to start Option B+. The HCWs explained that the clinics assigned a certain number of HCWs to work but not all assigned HCWs came to work at the same time. Community nurses go out to conduct outreach clinics in the communities on certain days of the week. HCWs sometimes go out for days or weeks to attend workshops or training, while others are on vacation. HCWs said clinics did not have enough HCWs to manage the large number of clients properly on most days, which we observed in the health facilities we visited.

*After the two [women] left, the nurse complained that she was tired and hungry but she did not want to stop because there were still women waiting outside*. **Field notes, 08-11-2015**

*“Eh, there is few staff, there are many people. For example, yesterday I was alone at antenatal. But there is need that at antenatal we should be more people… Actually yesterday, I had two allocations. I was supposed to work at antenatal and I was supposed to work at family planning. So, because at family planning… there was no Deprovera and other drugs, other contraceptives, right! So we did not do family planning … That is why I only did antenatal.”* **28 -year-old Female RN, SS04**

Shortage of staff reduced quality of care for Option B+ clients in three ways: a) it lowered quality of counseling, b) impeded HCW-client communication, and c) lengthened the time patients spent at the clinic.

### Quality of counseling

HCWs (16/35) admitted they gave their Option B+ clients suboptimal care. They explained that initial one-on-one counselling was short because they were tired and labored under time constraints. They rushed through sessions so they could see every woman waiting. Some HCWs felt this type of counseling insufficiently prepared Option B+ clients for a lifetime on ART because they skipped some required topics and they could not give clients enough time to process information or ask questions. HCWs assumed that the required group counseling for clients and support partners would somehow address the missing topics, but some HCWs realized that clients in group counseling were not free to ask personal questions. Others thought these topics would be tackled during ongoing counseling if clients remained in care, or that nurses could rely on ECs to fill the gaps.

*“…counselling is a big procedure on its own. The counseling rules requires 30 minutes to 1 hour because it is something that you need to explain so they understand but if you are there, you cannot have even 10 minutes to explain to somebody. That means you can only see two people in a day. So, people are not counseled in a proper way… Very fast. Generally, it’s because of the workload.”* **30-year-old male RN, SSI07**.

*“Initiation [on ART] differs with a subsequent client that started way back because at initiation you have to explain each and everything for the woman to understand. Most of the times group counseling, people say that it’s not effective because everyone has her own reasons, grievances so if you generalize that is when they do not understand”* **28-year-old female NMT, SSI20**

*“Fortunately it happens that… when we do not have many initial [first ANC visit] clients for ART it is possible but when we have many ART initial ANC clients mm… luckily we have the ECs that help with the counselling. Otherwise, if we did not have them it would have been a problem. The quality of service.”* **26-year- old female RN, SSI22.**

We saw that ongoing counseling sessions for clients on subsequent visits were much shorter.

*A woman came in for both antenatal and a refill of her ARVs. I heard the nurse ask her about her husband because he had tested negative when they tested together. I followed her [the nurse] to another room because the room we were in was crowded…. She gave her the ARV drugs and Bactrim then the woman left. This took about 10 minutes.* **Field notes, 14-08-2015**

### Poor HCW- client relationship

During our observations, we sometimes heard HCWs shouting at women who did not obey their requests.

*This one who was doing the weighing was shouting at the women because they were not responding or understanding as quickly especially when she asked them to stand by the wall for height measurement. The other nurse was also annoyed with someone because she said she was breathing close to her mouth. She also shouted at someone else because she was making her repeat questions. She told her that if she has to repeat every question then they will take so long to finish.* **Field notes, 08-12-2015**.

A few HCWs (4/35) said that HCWs sometimes shouted at women, perhaps because the women did not obey instructions, and that the HCW then punished the women by sending them to the back of the queue. This inconvenienced the women by lengthening their visits.

*“The other problem is that right here at the hospital the nurses shout and insult the clients and they do not talk to them properly. Maybe she missed [appointment date] after she was told to come on 15 December, right? It happens that the person has come on 20^th^ there is shouting for those [missed] days. “Did you not know that you were supposed to be here on such a date? So you have to sit there first; we have to help your friends who have come on their right dates”. Therefore, this at times makes the person disappointed, the person is already on ART, and they say aah! All these people, I should be behind them!”* **37-year-old Male EC, SSI33.**

HCWs also admitted shouting because they were fatigued and overwhelmed.

*“…sometimes you just find that maybe you can shout for things that do not necessarily require shouting but because you are tired. Someone is coming late you can turn them back and yet that is not allowed.”* **40-year-old Female RN (in-charge), SSI13**.

### Long wait time

#### First antenatal visit

HCWs (18/35) confirmed that women often spent long hours at the clinic. We observed that women spent between two and nine hours at the ANC during its opening hours. Women’s first antenatal visit took longer than later visits because it comprised several more steps (vaccinations, pre- and post-HIV/AIDS counseling, HIV testing, results then PMTCT). Women on this first visit went through these steps together (except for individual counseling) and finished together as a group.

*“…women on their initial visit (initials) have so many processes to go through, from Tetanus Toxoid Vaccine (TTV) they enter in here where we start our counseling related to antenatal. When we finish they should go in HTC where there is also their own counseling; then they test them one by one. Which means the queue for women on their subsequent visits… now women on their subsequent visits were done some time ago whereby the initials have not gone into HTC yet”* **28-year-old female NMT, SSI20**.

#### HCWs start work late

Sometimes women waited longer because the HCWs tasked to do HTC started work late or there was no one to conduct HTC. At some health facilities, this task was assigned to Health Surveillance Assistants (HSAs) whose main role is to provide community health care, but have been assigned to do this added work.

*“I think you are aware that most HTC counselors are HSAs… now they have… they have their priorities with their cadre. This is just a supplementary. Therefore, because of that they have gone for other campaigns. If the HTC office is not operational, what do you do? We send them back”* **42-year-old male EC, SSI16**.

We saw that HTC started late, and overheard a woman complain she had been turned away the day she came to have her child tested.

*The HSA who is also the counselor came in at 9*.*35 AM*. *He also works at the hypertension and diabetic clinic*, *which is where he was before he came*. **Field notes, 15-07-2015**

*…another woman came with her baby to give a dried blood spot sample*. *I heard her explain that she came the week before but was turned back because there was no [HTC] counselor to take the sample*. **Field notes, 14-08-2015.**

Nurses were frustrated at these delays. During one of our visits to a health facility, we saw an HSA leave the clinic before he finished testing all the women, which left no one to finish the task.

*An EC came to report to the nurse that the HSA had left. She said he had said that he was tired and left. He left when there were some women left to be tested and she said the women were complaining. The nurse said she was disappointed because she thought she would finish soon. She was alone doing both family planning and antenatal. Yesterday there was no counselor and today when she is about to finish, she had to go look for someone else to fill in. She looked frustrated.* **Field notes, 24-07-2015**.

#### Tools to motivate women during long waiting times

HCWs used tricks to encourage women waiting not to leave. For example, HCWs held on to women’s health passport books until the end of the clinic. In some clinics, the nurses waited to distribute mosquito nets until the end of the clinic.

*“The books remain with us… so the woman has come here for antenatal she cannot go home without the book. They wait. You find that at the end when we are handing them out… after teaching them we pile all the books. You find that everybody has collected their books. We call out the names as they are on the line with the book in our hands which means nobody can run away”* **39-year-old female community nurse, SSI09**.

*“Like how it happened yesterday, right! She [nurse] came up with a way of making the women stick around. [Laughs] She told them to wait and receive mosquito nets. So, I believe there was no one who left, eh! She just said wait here so that… You should get your nets because I am alone, I want that when we start distributing nets it should just be doing that so that everyone can be on their way. Therefore, the women stayed. They do talk, they do complain right!”* **27-year-old female EC, SSI26.**

To manage the staff shortage, nurses delegate some tasks to student nurses in teaching health facilities, or delegate to HSAs and ECs.

*“Like currently we can say that maybe we are better off because we have the students, right? Whom we have been with for a long time and they know most of the things. We supervise them on some things, right! That means the workload is small. However, if they are not there, there can be two people and we have to do routine antenatal, booking, palpations, then PMTCT and family planning. So there is a lot of work…”* **36-year-old female NMT, SSI29.**

*“…sometimes when we are called for some workshops, [nurse] may remain alone. It is not possible with how the clinic cohort is… For her to do it on her own… the good thing is all of them [ECs] did the ART training so it’s like they are capable of dispensing, like the first line drugs.”* **30-year-old male RN, SSI05.**

### Infrastructure limitations

#### Unconscious disclosure

HCWs (22/35) admitted that the limitations of the health facilities sometimes made it hard for them to conceal their client’s HIV status from other clients. In some health facilities, nurses escorted newly diagnosed HIV-positive women to the ART clinic to collect ARVs, and the women waiting on the benches outside could guess the HIV-status of the escorted women since the ART clinic only served HIV-positive clients. In other health facilities, nurses collected ARVs from the ART clinic and stored them in a basin or carton in the ANC room. Each time the nurses encountered an HIV-positive woman, they would cross the room to collect the ARVs in the basin and bring it to the client’s bed. Since there was often more than one client in the ANC room, other people saw what was in the basin.

*“That is one problem that we have. It is difficult… the privacy is just shielding the eyes only, so that maybe they should not see. But some do see because for you to get out and get the ARVs in the carton we keep them… people will still know that something is wrong. So we still do it because there is no other place to do it…”* **31-year-old female RN (in-charge), SSI03.**

*“… I give the counseling just between the two of us. Then I give her the ARVs. Then I go there [ART clinic] to register her so when they see her in front [of me], all the others clients know right away that the client is on ART. Therefore, confidentiality is a problem.”* **32-year-old female NMT, SSI34**.

Only two ANCs we visited had PMTCT rooms. HCWs almost always offered antenatal and PMTCT services in the same room. Most HCWs admitted at least two women to the room. When there were enough staff members, there would be two nurses and two clients. However, if there was only one nurse, she would talk to one woman while the other waited on the other bed. Under these conditions, if a nurse discovered the client was HIV-positive, she shouted to the next woman in line outside that she should not come in when the next woman left. HCWs knew that the women waiting would wonder why one woman was treated differently, and perhaps guess her HIV status.

*“…both are in since we are booking… one nurse has realised that her client is HIV positive, they can say that no one else should come in. This is still another thing not right. It is not good because with the reason that ‘um, why is that one taking time.’ Showing that, confidentiality can still be what … people can still realise that ‘there is something going on in there, then there must be something going on, why aren’t they allowing two people to go in there at the same time. Of course the confidentiality is supposed to be there but still a single room having to cater for two services at the same time brings thoughts that why are those taking time in there.”* **40-year-old female community nurse, SSI28**.

In some cases, we saw HCWs offering HIV/ART counselling in hushed tones in an ANC room full of HCWs and clients. Not having a room dedicated to PMTCT lowered the quality of counselling. HCWs kept counselling brief to minimize the risk that people would overhear.

*I could hear on the next bed that a client needed PMTCT counselling. The student nurse called the nurse in-charge to come and do it. She was counseling her in hushed tones but I still heard what was going on even though I was standing by the second bed. Then she went through the curtains and got the carton of ARVs. The room was not private at all because the three beds were close to each other and it was one room with at least 14 people going in and out.* **Field notes, 19-10-2015**.

*“…when a person is HIV positive, at least the person has to deliberate for a long time so as to accept it. However, considering the place we have… it is not well covered such that all the things you are discussing they can hear and they can see… we should just do brief counselling. I should just tackle only important points. Maybe the person is still in denial… it is also hard for the person to understand what you are saying, so I think to that issue concerning the place, it’s hard, there is need to have a special place.”* **31-year-old female RN (in-charge), SSI03**

### Clinics not organized to encourage husband attendance

HCWs (9/35) believed that their clinics were not set up to comfortably manage women and their husbands or offer security to their bicycles. Some clinics had a bench or two assigned in front of the line for couples while in some facilities HCWs said there was no place for husbands to sit. In some clinics, husbands isolated themselves; an observation that HCWs thought meant the husbands did not want to mingle with the women. None of the husbands we observed participated in any of the preliminary activities like the singing that took place before health education, nor did we observe the husbands responding to questions during the health talk.

*“Yes but still the infrastructure is not male-friendly. You see it can happen that they do not find anywhere to sit, the men just stand. Many men can come but they just wait [outside] looking after their bicycle because they see that security is not good that they can steal their bicycle. They just wait there [outside]. They do not get here. The ones that come here have nowhere to sit because the place is filled with women so for you to say women move for the men to sit, you still see that you are offending them”* **38-year-old female community nurse, SSI19**.

*At the end of the talk she asked the women if there are those who came with their husbands. There were only three. She explained that they get first priority. The men were not sitting on the foyer with the women. I saw one leaning against the next building behind the nurse.* **Field notes, 08-11-2018.**

### Cultural and gender barriers to accepting and adhering to ART

Despite HCWs’ belief that clinics were not organized to accommodate men well, HCWs encouraged women to come with their husbands because they believed husbands play a crucial role in women’s acceptance of ART, but most women we saw came alone. HCWs (20/35) told stories about how women refused or stopped treatment because they feared their husbands would leave them.

*“She said, “the problem is you can force me to take the drugs, I will get them, but I will not be taking the drugs. So it’s better you give them to other deserving women who can take these drugs”… third visit, we explain to her but she still says no… she cannot get the medicines because when her husband sees the drugs that means the marriage will end and the child will suffer. We told her that we want to protect her child… She says” you are just saying that because you do not know my husband. He cannot even come to the hospital to get a test”. So, what could we have done?.***” 32-year-old female NMT, SSI34**.

*“When she came back people discovered that she was a defaulter. She stopped taking the ARVs and she did not hide her problem. She explained, -“I took the drugs home and my husband denied me. He should not see me taking them in his house otherwise, I must go and stay with my mother and take my medicine there. What could I have done? Because I wanted marriage, I stopped”-”* **28-year-old female NMT, SSI20**.

HCWs still encouraged women to disclose to their husbands. To ease their fear of disclosure, HCWs gave women a note for their husband, requesting he attend the clinic without explaining why.

*“The reason is that they should get tested together since they have failed to reveal the results to their husbands on their own so they come so that they should hear the results together.”* **36-year-old female EC, SSI32**.

Women accompanied by husbands on their first or later ANC visits received quick service. HCWs tried to move them through the steps as fast as possible, making their waiting time much shorter than that of individual women, who went through these steps as a group and had to wait for each other.

*“But when they come as a couple, those that come with their husband, we help them right there. They will go for testing very quickly, the same time they are going for weighing, the same time they go for palpation of their pregnancy, the same time they are given a mosquito net, same time they are going. At least maybe 1 hour”* **38-year-old female community nurse, SS017**

## Discussion

This study explored health system barriers to Option B+. The main barriers HCWs faced in providing high-quality patient care to HIV-infected pregnant and lactating women on Option B+ include staff shortages that lowered quality of care, strained HCW-client relationships, and lengthened clients’ visits to the clinic. They also faced difficulties posed by infrastructure that did not allow them to keep consultations with clients private. HCWs were concerned that clinics were not organized to encourage male attendance. Culture and gender also affected women’s initiation on ART or their adherence.

Malawi’s health care system suffers from inadequate human resources. [2,19,29] According to the Malawi health sector strategic plan II, the public health sector is operating at a 45 percent vacancy rate [19], less than WHO requirements. [6] This confirms our finding that health facilities are understaffed posing a serious challenge to proper implementation of Option B+, a finding consistent with other studies. [5,14,23,30] For example, Tenthani et al. found that overburdening HCWs may contribute to high LTFU of women in health facilities with heavy patient load. [20] Like Bradley et al. [31], we found HCWs were overburdened. Their patient loads were so heavy that they had to cut counseling sessions short, which they believed reduced its effectiveness and the likelihood it would address patients’ psychosocial needs. Since initial counseling can help women accept ART, build relationships of trust with HCWs, and improve ART adherence, this is a matter of serious concern. [20,32,33] HCWs thought that the gaps left by these brief sessions is captured by group counseling and counseling on later visits. From our observations, this did not appear to be true. This agrees with findings from some women disengaged from care who felt their initial counselling was inadequate [34]. Since studies have found counselling can motivate women on Option B+ to stay in care, the short sessions caused by overburdened staff could have detrimental effects on the program as a whole [17,34]. The speed of a woman’s induction into Option B+ can also be coercive when it does not give her time to deliberate [20,32,34], which may be why same-day initiation has been associated with LTFU. [30,35,36] Women who had more time to deliberate, who received additional counseling, and who initiated ART later had better retention. [20]

Other studies have highlighted long waiting times as a barrier to Option B+. [23,37,38] We saw that long waiting times are hardest on all women on their initial antenatal visit however the wait is longer for newly diagnosed HIV positive women. HCWs withholding benefits to make women stay until the end of clinic works in the short term, but is not a sustainable solution to the underlying problem, especially for women who cannot afford to be away from their income generating activities for long periods. [39]

Previous studies have reported on poor communication between women and HCWs, and identified this as a reason some patients disengage from care. [5,22,33,37] Encouragement from HCWs has been associated with more positive outcomes for Option B+. [12,37] In our study, HCWs shouted because they were tired. Flax et al. suggest that women may tolerate abusive communication from HCWs because they do not think they have the power to change it. [22] None of the women we saw on the receiving end of this poor communication ventured a response, which may align to Flax et al.’s argument.

The layout of the health facility and its lack of space made it difficult for HCWs to protect women’s confidentiality under Option B+. [30, 38] It was almost impossible to conceal client’s HIV status at most of our study sites. Although health facilities have strict rules protecting patients’ privacy, the rules are difficult to follow if the facilities offer no privacy. In some facilities, clients were escorted to ART clinics, a study in Tanzania said was as good as walking with a title on your back saying, “I am HIV positive.” [38] HCWs realized they breached clients’ confidentiality, but felt they had no better options. Some HCWs thought the situation could be improved if there was a special room reserved for PMTCT, but we doubt this, since other studies found that clients seen queueing or going to the ART clinic were still publicly labeled HIV-infected. [5,14,22,30,38] This is why many women who feel compelled to conceal their HIV status prefer walking long distances to access ART at a health facility where they are unlikely to meet anyone they know. [14,38,40] Though we and Chan et al. [35] found that HCWs believed women feel less threatened if they received ART at an ANC clinic where services were integrated, we observed that ANC infrastructure also could not provide privacy due to the space limitation.

Results from other studies show that support from husbands and partners is crucial to the success of Option B+ [5,22,30,37,41]. Some studies have shown that couple HIV-test yields better acceptance and adherence to ART in pregnant and lactating women [17,37,42,43] similar to our findings. HCWs in our study described cases of women refusing or stopping treatment because their husband threatened to divorce them after they informed them of their HIV positive test result. Though HCWs encouraged male involvement, they did not think the clinic was set up to encourage male participation, a finding that concurs with other studies. [41,44,45] Menjate et al in their review found that men may be uncomfortable sitting close to women they don’t know in crowded clinics [46] or in an environment dominated by women. [45,46] Other studies in Malawi found male involvement in pregnancy can be limited by traditional gender roles, where the common belief is pregnancy is women’s business [22,44,45,47]. Some men fear that other men will question their masculinity or stigmatize them if they accompany their wives to ANC, and worry it will look like their wife controls them. [22,45,46] Some men have reported feeling excluded at clinics, because ANC focuses on the woman [46], or do not consider activities at the clinic, like singing and dancing, to be appropriate for men. [44]

It is impossible to separate gender issues in HIV treatment from gender issues in the larger society. Malawi ranks 173 out of 188 countries on the UN’s Gender Inequality Index. [48] This is why interventions that presume equality, like telling women that they should ask their partners for condoms, or forcing women to disclose to men who have economic power over them, or insisting women bring their male partners to the clinic, so often fail. Changing that imbalance is crucial to eradicating HIV in the long-term, but in the short term, interventions that do not require upending existing power structures can be effective. Since men are more likely to follow directions from HCWs than from their wife [41,43], health authorities can give women notes that request their husband’s presence. [43,45] Or local chiefs can enact bylaws that require every man to escort their pregnant wife to ANC [44].

Our study was limited because we were not able to visit facilities in the northern part of the country, so our results may not be generalizable to Malawi’s northern region. But it is worth noting that all health facilities in Malawi follow the same PMTCT guidelines. Our study was however strengthened by our use of complementary data collection methods (SSIs and observations) that helped us triangulate the perspectives of HCWs. We could probe HCWs on specific points we observed, which worked particularly well to address the question of unconscious disclosure, which might not otherwise have been revealed as a health system barrier.

## Conclusion

The HCWs implementing Option B+ are so overburdened in Malawi that it reduces their ability to provide quality care to clients, who receive less counseling than they should, wait longer than is reasonable, and have very little privacy. Interventions that increase the number of HCWs could improve quality of care. Malawi’s health system recognizes this problem as evidenced by the deployment of HIV diagnostic assistants in 2015 that has improved HIV and other diagnostic testing. [49] This solves part of the problem. There is need for more interventions. Upgrading the infrastructure to protect the privacy of HIV-infected pregnant and lactating women and their husbands could increase retention, but facilities will need improvement to support men who accompany their partners on clinic visits.

## Supporting information

S1 Text (DOCX)

S2 Text (DOCX)

S3 Text (DOCX)
