# Supplementary material for: “I do all I can but I still fail them”: Health system barriers to providing Option B+ to pregnant and lactating women in Malawi
Source: PLoS One. 2019 Sep 12;14(9):e0222138. doi: 10.1371/journal.pone.0222138 (PMC6742345; doi:10.1371/journal.pone.0222138)
Supplement: S1 Text — (DOCX) [file pone.0222138.s001.docx]

# **SEMI-STRUCTURED INTERVIEW GUIDE**

## **Socio-demographic data**

Collect the following socio-demographic details from all the participants at the beginning or at the end of the interviews.

- Age, marital status, number of children, occupation, position and religion.

Respondents will drive the semi-structured interviews. However, at a minimum, try to explore the following questions/issues in detail with the healthcare workers during the interviews. Also, explore any other relevant issues that will emerge during the discussions or that you noticed during your ethnographic observations.

1. Tell me more about your daily work at the hospital.

***Probe:*** *what do you do when you get to work until you knock off?*

1. How many clients/patients do you assist in a day?
2. How do you assist clients/patients when they come to your office?

***Probe:*** *how do you assist Option B+ clients?*

1. How many staff are there in your department/section/unit

***Probe:*** *Do you think that the staff is enough?*

1. Which other sections/departments do the clients/patients go (or both first time and follow-up visits)?

***Probe:*** *are things different for first time clients than for clients on subsequent visits?*

1. What challenges do you encounter when you deal with clients (both technical and social)?

*Probe for specific challenges from your observations*

1. How do these challenges affect the way you treat your clients?
2. What do you think needs to be done to address these challenges with your clients?
3. What administration or management challenges do you face in your work?
4. How do these challenges affect the way you discharge your duties?
5. What do you think needs to be done to address these challenges?
6. From your perspective, what are the barriers to Option B+?
7. What facility level issues do you think influence client`s decision not to return for Option B+ (loss to follow-up)?
8. What can be done at facility/community/client level to improve Option B+ retention?
