# Supplementary material for: “I do all I can but I still fail them”: Health system barriers to providing Option B+ to pregnant and lactating women in Malawi
Source: PLoS One. 2019 Sep 12;14(9):e0222138. doi: 10.1371/journal.pone.0222138 (PMC6742345; doi:10.1371/journal.pone.0222138)
Supplement: S2 Text — (DOCX) [file pone.0222138.s002.docx]

# Observational method

Our observations in facilities will be at three different levels even though the lines separating these levels are not clear: (1) facility; (2) individual; and (3) interactional levels. At facility level, focus will be on the general structure of the facility and specifically the set-up of all the sections that are part of the Option B+ services. Our interest is on things like waiting space, number of staff and availability of equipment and drugs among others. At individual level, we will follow some clients (clients on their initial or subsequent visits) from the time they arrive at the facility up to the time they leave. The idea behind is to understand all the processes and or stages that clients who are under the option B+ go through at the facility. Emphasis will be given to the total time spent at the facility, time spent at each station, the type of care or treatment they receive and who exactly accompanies them. Since observational method can also involve asking questions where necessary, we will briefly ask health care workers informally where it is not clear. In addition, we are also interested in the client – clinician interaction at the facility. As such, we will observe as much interactions as possible between the two. We will focus on the mood, feedback, content and specific words used among others. This will help us to understand the relationship between the two and how that might affect uptake and retention.

We will generally do the observations without taking any detailed notes to avoid distraction. However, we will jot down important things that we can easily forget during the course of observations though we will keep this as minimal as possible. We will make detailed notes of the observations before the end of each field day while things are still fresh in the memory of the observer.
