# Supplementary material for: “I do all I can but I still fail them”: Health system barriers to providing Option B+ to pregnant and lactating women in Malawi
Source: PLoS One. 2019 Sep 12;14(9):e0222138. doi: 10.1371/journal.pone.0222138 (PMC6742345; doi:10.1371/journal.pone.0222138)
Supplement: S3 Text — (DOCX) [file pone.0222138.s003.docx]

**“I do all I can but I still fail”: Health system barriers to providing Option B+ to women in Malawi**

**Codebook**

| **Codes** |  |
| --- | --- |
|  |  |
| **Shortage of staff** | **Definition:** Not enough health care workers to do the required job well. The number of health care workers allocated to a clinic versus actual number working.    **Categories:**  **Quality of counselling**- Delivery of sub- optimal counselling.  **Health care worker- patient communication-** Abusive talk, shouting, or treatment by health care workers to patients.  **Long waiting time**- lengthened stay at the clinic from time the patients arrive to when they finished and left the clinic. |
| **Infrastructure limitation** | **Definition:** Limited space or rooms in the clinics.    **Categories:**  **Unconscious disclosure-** Any unintended disclosure of patients’ HIV status to other parties. |
|  |  |

| **Clinic setup discouraging male involvement** | **Definition:** Health care workers’ belief that the clinic setup failed to accommodate men by either making them feel uncomfortable or not able to provide a place to sit. |
| --- | --- |
| **Cultural –gender barrier** | Stories of women who struggled initiating or staying on ART because of community or social interaction such as fear of divorce from the husband or husband demand to stop ART. |
